# Supplementary material for: Acoustic shadows help gleaning bats find prey, but may be defeated by prey acoustic camouflage on rough surfaces
Source: eLife. 2015 Sep 1;4:e07404. doi: 10.7554/eLife.07404 (PMC4550812; doi:10.7554/eLife.07404)
Supplement: Supplementary file 1. — Additional statistical details referring to all tests. DOI: http://dx.doi.org/10.7554/eLife.07404.020 [file elife07404s007.docx]

**Supplementary File 1:** Additional statistical details referring to preferred models.

| **Table S1a Term** | **Estimate** | **St. error** | **d.f.** | **t-value** | **p-value** |
| --- | --- | --- | --- | --- | --- |
| Intercept | 4.0153 | 0.1467 | 108 | 27.3793 | **0.0000** |
| Wing | -0.0773 | 0.0336 | 108 | -2.3022 | **0.0232** |
| Substrate (leaf) | 0.4322 | 0.0949 | 108 | 4.5523 | **0.0000** |
| Substrate (limestone) | 0.6195 | 0.0949 | 108 | 6.5258 | **0.0000** |
| Substrate (slate) | 1.6527 | 0.0949 | 108 | 17.4087 | **0.0000** |
|  |  |  |  |  |  |
| **Table S1b Term** | **Estimate** | **St. error** | **d.f.** | **t-value** | **p-value** |
| Intercept | -5.4024 | 3.0727 | 105 | -1.7582 | 0.0816 |
| Wing | -1.4104 | 1.2893 | 105 | -1.0939 | 0.2765 |
| Substrate (leaf) | -10.8977 | 4.0771 | 105 | -2.6729 | **0.0087** |
| Substrate (limestone) | 14.1890 | 4.0771 | 105 | 3.4802 | **0.0007** |
| Substrate (slate) | -30.9086 | 4.0771 | 105 | -7.5811 | **0.0000** |
| Wing* Substrate (leaf) | 0.3713 | 1.8233 | 105 | 0.2037 | 0.8390 |
| Wing* Substrate (limestone) | -4.5058 | 1.8233 | 105 | -2.4712 | **0.0151** |
| Wing* Substrate (slate) | -0.1295 | 1.8233 | 105 | -0.0710 | 0.9435 |
|  |  |  |  |  |  |
| **Table S1c Term** | **Estimate** | **St. error** | **d.f.** | **t-value** | **p-value** |
| Intercept | 4.0153 | 0.1467 | 108 | 27.3793 | **0.0000** |
| Wing | -0.0773 | 0.0336 | 108 | -2.3022 | **0.0232** |
| Substrate (leaf) | 0.4322 | 0.0949 | 108 | 4.5523 | **0.0000** |
| Substrate (limestone) | 0.6195 | 0.0949 | 108 | 6.5258 | **0.0000** |
| Substrate (slate) | 1.6527 | 0.0949 | 108 | 17.4087 | **0.0000** |
|  |  |  |  |  |  |
| **Table S1d Term** | **Estimate** | **St. error** | **d.f.** | **t-value** | **p-value** |
| Intercept | -1.3050 | 0.0709 | 556 | -18.412 | **<2x10^-16^** |
| Angle | 0.0183 | 0.0018 | 556 | 10.472 | **<2x10^-16^** |
| Substrate (leaf) | -0.4682 | 0.1002 | 556 | -4.671 | **3.8x10^-06^** |
| Substrate (limestone) | -0.2090 | 0.1002 | 556 | -2.085 | **0.0376** |
| Substrate (slate) | -0.1399 | 0.1002 | 556 | -1.395 | 0.1635 |
| Angle*Substrate (leaf) | -0.0071 | 0.0025 | 556 | -2.853 | **0.0045** |
| Angle*Substrate (limestone) | -0.0043 | 0.0025 | 556 | -1.753 | 0.0801 |
| Angle*Substrate (slate) | -0.0252 | 0.0025 | 556 | -10.176 | **<2x10^-16^** |
|  |  |  |  |  |  |
| **Table S1e Term** | **Estimate** | **St. error** | **d.f.** | **t-value** | **p-value** |
| Intercept | -1.2103 | 0.0190 | 556 | -63.850 | **<2x10^-16^** |
| Angle | 0.0162 | 0.0005 | 556 | 34.518 | **<2x10^-16^** |
| Substrate (leaf) | -0.4637 | 0.0268 | 556 | -17.299 | **<2x10^-16^** |
| Substrate (limestone) | -0.2281 | 0.0268 | 556 | -8.510 | **<2x10^-16^** |
| Substrate (slate) | -0.4418 | 0.0268 | 556 | -16.479 | **<2x10^-16^** |
| Angle*Substrate (leaf) | -0.0020 | 0.0007 | 556 | -3.009 | **0.0027** |
| Angle*Substrate (limestone) | -0.0031 | 0.0007 | 556 | -4.676 | **3.7x10^-6^** |
| Angle*Substrate (slate) | -0.0085 | 0.0007 | 556 | -12.839 | **<2x10^-16^** |
|  |  |  |  |  |  |
| **Table S1f Term** | **Estimate** | **St. error** | **d.f.** | **t-value** | **p-value** |
| Intercept | -8.7239 | 0.0545 | 556 | -160.080 | **<2x10^-16^** |
| Angle | -0.0095 | 0.0013 | 556 | -7.085 | **4.2x10^-12^** |
| Substrate (leaf) | 0.2981 | 0.0771 | 556 | 3.868 | **0.0001** |
| Substrate (limestone) | -0.2233 | 0.0771 | 556 | -2.898 | **0.0039** |
| Substrate (slate) | -0.2057 | 0.0771 | 556 | -2.669 | **0.0078** |
| Angle*Substrate (leaf) | -0.0177 | 0.0019 | 556 | -9.323 | **<2x10^-16^** |
| Angle*Substrate (limestone) | -0.0065 | 0.0019 | 556 | -3.431 | **0.0006** |
| Angle*Substrate (slate) | 0.0008 | 0.0019 | 556 | 0.404 | 0.6867 |
|  |  |  |  |  |  |
| **Table S1g Term** | **Estimate** | **St. error** | **d.f.** | **t-value** | **p-value** |
| Intercept | -8.719 | 0.0593 | 556 | -146.973 | **<2x10^-16^** |
| Angle | -0.0101 | 0.0015 | 556 | -6.892 | **1.5x10^-11^** |
| Substrate (leaf) | 0.4194 | 0.0839 | 556 | 4.999 | **7.8x10^-07^** |
| Substrate (limestone) | -0.2673 | 0.0839 | 556 | -3.186 | **0.0015** |
| Substrate (slate) | -0.2550 | 0.0839 | 556 | -3.040 | **0.0025** |
| Angle*Substrate (leaf) | -0.0224 | 0.0021 | 556 | -10.827 | **<2x10^-16^** |
| Angle*Substrate (limestone) | -0.0092 | 0.0021 | 556 | -4.436 | **1.1x10^-5^** |
| Angle*Substrate (slate) | 0.0012 | 0.0021 | 556 | 0.597 | 0.5509 |
